# Adolescent Reports of Demanding and Responsive Features in Helicopter and Overprotective Parenting: Divergence of Correlations With Indicators of Adjustment

**DOI:** 10.1002/jad.70218

**Published:** 2026-07-09

**Authors:** Melanie J. Zimmer‐Gembeck, Katherine M. Ryan, Bart Soenens, Grégoire Zimmermann, Stijn Van Petegem

**Affiliations:** ^1^ School of Applied Psychology Griffith University Southport Queensland Australia; ^2^ Griffith Mental Health Research Centre Griffith University Southport Queensland Australia; ^3^ Department of Developmental, Personality, and Social Psychology Ghent University Ghent Belgium; ^4^ Family and Development Research Center (FADO), Institute of Psychology University of Lausanne Lausanne Switzerland; ^5^ Universite Libre de Bruxelles Brussels Belgium

**Keywords:** adolescent adjustment, coping skills, decision making, helicopter parenting, overprotective parenting, parenting behaviors

## Abstract

**Introduction:**

Research collecting data from parents has found that helicopter parenting (HP) and overprotective parenting (OP) of their adolescents can be reformed to identify two features: demanding and responsive overparenting (Ryan et al. 2024). The correlations of these features with other measures were also found to differ, with demanding overparenting uniquely correlated with parent reports of less positive parenting and poorer youth adjustment and with responsive overparenting correlated with more parent‐reported positive parenting. We aimed to determine if these features are also found when parenting is reported by youth and to validate the features by correlating them with youth experiences of positive parenting and youth self‐reports of their adjustment.

**Methods:**

Australian adolescents aged 16–19 years (*N* = 512; 34% boys, 64% girls, 2% nonbinary) completed surveys to report HP, OP, received parental support, psychological distress, decision‐making, and coping efficacy. Data were analyzed with confirmatory factor analysis and partial correlations.

**Results:**

Demanding and responsive features of overparenting were confirmed. When controlling for shared variance between the features, demanding overparenting was associated with less but responsive overparenting was associated with more parental support. Demanding overparenting was associated with higher, whereas responsive overparenting was associated with lower, youth psychological distress. Correlations also differed for youth's decision‐making and coping.

**Conclusions:**

The findings are consistent with the idea that measures of intrusive parenting can be differentiated into a more coercive form—referred to as demanding overparenting—from a form that may be in response to tasks and decision‐making—referred to as responsive overparenting.

1

In the past two decades, helicopter parenting (HP) and overprotective parenting (OP)—which capture parenting practices that are overinvolved and autonomy‐restrictive of adolescents and emerging adults—have attracted a great deal of research and popular media attention. This research tends to find that HP and OP are associated with less mature levels of autonomy and agency (Flamant et al. 2022; Jiao and Segrin 2023) and with less positive psychological adjustment (such as more depressive symptoms) among adolescents and emerging adults (see Ryan et al. 2024 and Zhang and Ji 2024, for reviews). HP has been described as parents' overinvestment in helping children to meet goals and achievements (Cline and Fay 1990), with parents restricting their children's autonomy and feelings of competence by hovering over their children (Buchanan and LeMoyne 2020; Cook 2020; Lindke and Oppenheimer 2022) or being excessively protective (Gomes and Deuling 2019; Hwang and Jung 2022; Kwon et al. 2016). OP has been defined as parenting that does not support the development of autonomy, agency, independence, or critical decision‐making skills representative of what would be expected for children of a given age (for a review see Cui et al. 2022), as intrusive and overcontrolling (Givertz and Segrin 2014; Leung 2021; Trbovich et al. 2022), as excessive shielding and overprotection (Chevrier et al. 2023; Zheng et al. 2019), and as excessive guidance, support and problem solving assistance (Gagnon et al. 2020; Segrin et al. 2015).

Consistent with the definitional overlap between HP and OP, they share some variance in empirical studies (e.g., Leung and Shek 2018; Segrin et al. 2012; Vigdal and Brønnick 2022). Yet, when measures of HP and OP are completed by parents, evidence has emerged that these measures can be used to reveal demanding and responsive features of overparenting. Ryan et al. (2024) collected reports from parents about their HP and OP using the Helicopter Parenting Instrument (HPI; Odenweller et al. 2014) and the Short Multidimensional Overprotective Parenting Scale (S‐MOPS; Chevrier et al. 2023), finding two emergent features of overparenting. Demanding overparenting (mostly indicated by items from the S‐MOPS) tapped more directly intrusive, autonomy‐restrictive, and coercive behaviors of parents. Responsive overparenting (made up of mostly items from the HPI) tapped parents' tendency to take over youth's management of personal and social activities, in response to challenging or stressful circumstances. Demanding and responsive overparenting were positively correlated with each other, as would be expected by their shared focus. However, correlations of parent‐reported demanding and responsive overparenting with other measures differed, particularly when their shared variance was controlled for. Demanding overparenting was uniquely related to less parent‐reported autonomy support and parental acceptance of the child, more rejection and coercion, more parent and child anxiety symptoms, more parental burnout, less enjoyment of parenting, feeling worse at parenting when comparing to other parents on social media, and more job demands. In contrast, responsive overparenting was related to more parent‐reported autonomy support and parental acceptance of the child, not significantly related to rejection, coercion, parent/child anxiety, parental burnout or job demands, and related to more enjoyment of parenting ‐ but responsive overparenting was also weakly associated with parental reports of comparisons to other parents on social media and with perceiving more pressure from others to be a perfect parent. It remains unclear, however, whether this differentiated pattern of associations would emerge also in youths' perceptions of parenting and adjustment.

Such findings align with other research that has suggested that HP (which contributed more items to the measurement of responsive overparenting) is controlling and restricting *but* also involves warmth and support by being responsive to youth's needs for assistance and emotional or practical support (Earle and LaBrie 2016; Kouros et al. 2017; Luebbe et al. 2018; Reed et al. 2016). In contrast, OP (which contributed most items to the measurement of demanding overparenting) has been described as centered on excessive restrictions and overprotection, with an aim to keep this distinct from parental warmth and support (Chevrier et al. 2023; Gagnon et al. 2020).

This study is the first to investigate if demanding and responsive overparenting features can be identified from youth reports of HP and OP. It is also the first to estimate the correlations of demanding overparenting and, separately, responsive overparenting with youth's (rather than parents’) report of parenting practices and personal adjustment, and to test the differences between these correlations. Focusing on reports from youth is notable given that many studies rely only on adolescents or emerging adults for information about their experiences of HP or OP. In addition, this is the first study to contrast associations of youth‐report of demanding versus responsive overparenting with a range of youth adjustment indicators, which have been the primary focus of research on HP and OP in the past (Ryan et al. 2026).

## 2 Methods

### 2.1 Participants

The study participants were 512 Australian adolescents 16–19‐years‐old (*M* = 17.4 years, SD = 0.6; 34% boys, 64% girls, 2% nonbinary). The youth were in their final year of high school (60.9%) or in their first 3 months of their first year of university after graduating from high school in the previous year (39.1%). The youth could select multiple responses for race/ethnicity and reported their birth country and language usually spoken at home. Overall, 71.7% reported White; 6.4% First Nations Australian, Torres Strait Islander or Pacific Islander; 17.6% Asian; and 13.9% other including African, Indian, Fijian, South American and multiple other backgrounds. More than three‐fourths (78.5%) reported they were born in Australia and 5.1% were born in New Zealand. Most (88.1%) spoke English at home.

## 3 Procedure

Following approval of the study by the Griffith University human research ethics committee (Protocol #2022/436) and the Queensland state education department, youth were recruited in two ways. First, incoming university students in the first 3 months of their first year were invited to participate through university research advertisements. Students at university provided their own consent to participate. Second, the study was advertised to all Year 12 students through research staff visits to 10 local secondary schools (i.e., schools with students in grades 7–12) and advertised in the newsletter of another seven local secondary schools. Parents could opt in to provide informed consent for their child or students were given a link to pass on to their parents to provide informed consent for their participation. Once informed consent was received, the secondary school student was given a link to complete their survey online. Assent for participation was required for youth participation, as well. The portions of the survey included in this study were completed in approximately 20 min. Youth were sent $20 electronic gift vouchers following their participation. Data are available upon reasonable request from the first author.

## 4 Measures

All measures have been used in past research and had good reliability and validity. To form subscale scores, relevant items were averaged for all measures with higher scores indicating more of each behavior.

### 4.1 Helicopter and Anxious Overprotective Parenting

Youth completed the Helicopter Parenting Instrument (HPI; Odenweller et al. 2014; e.g., “My parent tries to make all of my major decisions”) youth‐report version and the anxious overprotection scale of the Short Multidimensional Overprotective Parenting Scale modified for youth report (S‐MOPS; Chevrier et al. 2023; “My parent often takes over tasks without giving me the chance to try them myself first”). Response options for the HPI and the S‐MOPS ranged from 1 (*Very strongly disagree*) to 7 (*Very strongly agree*). Following confirmatory factor analysis and scoring procedures reported in past research for parent report versions of the HPI and the S‐MOPS (Ryan et al. 2024), two subscale scores were formed: demanding overparenting (four HPI items and nine S‐MOPS items) and responsive overparenting (eight items from the HPI). Cronbach's α and other details are presented in the Results.

### 4.2 Positive Parenting and Parental Support

Positive parenting, including warm interactions (three items) and support for autonomy (three items), was measured with the Parents as Social Context Questionnaire (PSCQ; Skinner et al. 2005; “My parent encourages me to be true to myself,” α = 0.90). Parental support was measured with the 10‐item parent subscale of the Parent and Peer Attachment Scale (PPAS; Armsden and Greenberg 1987; “I can count on my parents when I need to talk about something important”), Cronbach's α = 0.81. Response options for the PSCQ and the PPAS ranged from 1 (*No! Not at all true for me*) to 6 (*Yes! Totally true for me*).

### 4.3 Psychological Distress

Youth's psychological distress was measured with the 10‐item Kessler 10 Scale of psychological distress (Kessler et al. 2002; e.g., “In the past 4 weeks, about how often did you feel nervous?”). Response options ranged from 1 (*None of the time*) to 5 (*All of the time*), Cronbach's α = 0.92.

### 4.4 Coping Efficacy

Coping efficacy was measured with seven items (Sandler et al. 2000; e.g., “How satisfied are you with the way you handled your problems during the last month?”). Response options ranged from 1 (*Not at all*) to 4 (*Very good*), Cronbach's α = 0.86.

### 4.5 Decision‐Making

Youth's decision‐making strategies were measured using items from the General Decision‐Making Scale (Scott and Bruce 1995; “I make decisions in a logical and systematic way”). Subscales included rational (two items, *r* = 0.55), dependent (two items, *r* = 0.59), avoidant (three items, Cronbach's α = 0.86) and spontaneous (two items, *r* = 0.68) decision‐making. Response options ranged from 1 (*No! Does not describe me at all*) to 5 (*Yes! Describes me greatly*).

### 4.6 Data Analyses

AMOS v30 was used to confirm the two expected features of demanding and responsive OP. Correlations were estimated using SPSS v30 to report the associations between all measures. We used z‐tests to compare the strength of correlations of demanding and responsive OP with other measures. Finally, we estimated partial correlations. These correlations show the unique associations of one overparenting feature (i.e., demanding or responsive overparenting) with other measures (e.g., positive parenting) net of the other overparenting feature.

## 5 Results

### 5.1 Confirmatory Factor Analysis

Table 1 shows the results of the confirmatory factor analysis of the 13 demanding overparenting and the 8 responsive overparenting items. As can be seen, the model had an adequate fit to the data after freeing three covariances between demanding overparenting item errors and three covariances between responsive overparenting item errors. The loadings for demanding overparenting indicated a strong factor, loadings ranged from 0.49 to 0.82. The responsive overparenting factor appeared more weakly represented by the items, but still had loadings above 0.30 for all items with the highest loading of 0.73. Demanding and responsive overparenting items had Cronbach's α = 0.93 and 0.76, respectively.

**Table 1:** Results of the confirmatory factor analysis of helicopter parenting (HP) and overprotective parenting (OP) items (*N* = 512).

Items	Demanding OP	Responsive OP
All my major decisions (HPI)	0.82	
Involves themself in things (S‐MOPS)	0.81	
Treats me in a childish way (S‐MOPS)	0.80	
All over me (S‐MOPS)	0.80	
Too involved in my life (S‐MOPS)	0.78	
Violates privacy (S‐MOPS)	0.75	
End up in trouble (S‐MOPS)	0.75	
Takes over tasks (S‐MOPS)	0.74	
Sees danger (S‐MOPS)	0.74	
Treats like a small child (S‐MOPS)	0.69	
Discourages me from decisions (HPI)	0.61	
Insists that I inform them (HPI)	0.60	
Parent invests more time and energy (HPI)	0.49	
Overreacts to negative experience (HPI)		0.73
Step in and “save” me from difficulty (HPI)		0.59
Shield me from bad things (HPI)		0.58
Voices their opinion about relationships (HPI)		0.54
Solve problems (HPI)		0.48
Always tries to fix it (HPI)		0.39
Parent takes over tasks (HPI)		0.36
Parent accompanies me (HPI)		0.32

*Note:* χ^2^ (181) = 710.81, *p* < 0.001; CFI = 0.92, NFI = 0.89, RMSEA = 0.070, 90% CI 0.065–0.075, *p* < 0.001.

### 5.2 Comparing Correlations of Demanding and Responsive Overparenting With Other Measures

Demanding and responsive overparenting were highly intercorrelated, *r* = 0.68. However, as can be seen in Table 2, demanding and responsive overparenting scores had significantly different associations with multiple other measures. For the unadjusted correlations of demanding and responsive overparenting with other measures, three significant differences were found after using a Bonferroni correction to adjust the *p*‐value to < 0.006 to account for nine comparisons (0.05/9 = 0.006). Demanding overparenting was more highly correlated with youth's psychological distress than responsive overparenting. Also, demanding overparenting was negatively associated with positive parenting and parental support, whereas there were no significant associations of responsive overparenting with these positive parenting measures. When partial correlations were estimated, demanding overparenting (unique from responsive overparenting) stood out as significantly related to (*p* < 0.006) less positive parenting, and more youth psychological distress, less rational decision‐making, more avoidant and impulsive decision‐making, and less coping efficacy. In contrast, responsive overparenting (unique from demanding overparenting) was associated with more positive parenting and parental support and more dependent decision‐making but not significantly correlated at *p* < 0.006 with any other measures.

**Table 2:** Correlations (and partial correlations) of youth‐report demanding overparenting (OP) and responsive OP with all other measures, and results of testing differences (z‐score) between the dependent correlations (*N* = 512).

Measures	Mean	SD	Demanding OP, *r*	Responsive OP, *r*	Z‐score	Demanding OP partial *r* a	Responsive OP partial *r* b	
Demanding OP	3.30	1.24	—		—	—	—	
Responsive OP	3.84	0.95	0.68*	—	—	—	—	
Positive parenting	4.29	1.17	−0.37*	0.00	−10.80*	−0.50*	0.37*	
Parental support	4.09	1.05	−0.44*	−0.06	−11.32*	−0.55*	0.37*	
Parent academic pressure	3.17	1.46	0.33*	0.19*	4.12*	0.27*	−0.04	
Psychological distress	2.80	0.92	0.40*	0.23*	5.12*	0.34*	−0.07	
Coping efficacy	2.76	0.58	−0.19*	−0.09**	−2.86***	−0.18*	0.05	
Rational decision‐making	3.60	0.85	−0.17*	−0.08	−2.56***	−0.16*	0.05	
Dependent decision‐making	3.25	1.00	0.11**	0.21*	−2.87***	−0.04	0.18*	
Avoidant decision‐making	3.02	1.08	0.26*	0.21*	1.46	0.16*	0.05	
Impulsive decision‐making	2.69	1.05	0.21*	0.14***	2.01**	0.16*	0.00	

*Note:* Reporting of significant associations in the text was limited to those with *p* < 0.006 to adjust for multiple comparisons and partial correlations.

^a^ Correlation after adjusting for Responsive OP (*r*_Demanding OP, adjusting for Responsive OP_).

^b^ Correlation after adjusting for Demanding OP (r_Responsive OP, adjusting for Demanding O__P_).

* *p* < 0.001

** *p* < 0.05

*** *p* < 0.01.

## 6 Discussion

This study confirmed that youth's report of anxious overprotective and helicopter parenting can be reformed into demanding and responsive forms of overparenting, consistent with what was found in a study relying on parent report (Ryan et al. 2024). As found previously, demanding and responsive overparenting were positively correlated with each other in the current study, but also notably differed in their patterns of correlations with youths' perceptions of positive and supportive parenting and their psychological adjustment, general decision‐making abilities, and coping efficacy. These differential associations became more pronounced when controlling for the shared variance between the two features.

## 7 Positive Parenting and Youth's Psychological Distress

The findings for overparenting as related to positive parenting were particularly notable. Before and after adjustment for responsive overparenting, demanding overparenting was more strongly related to less positive and supportive parenting. Also, after adjustment for demanding overparenting, responsive overparenting was linked to more positive parenting and parental support. As previously found based on parent report, differentiating demanding and responsive features of overparenting may help to explain why measures of intrusive parenting, such as HP, are sometimes associated with more parental warmth and support, but are also described as related to more coercive or autonomy‐restrictive parenting in other studies.

Youth who reported receiving more demanding and responsive overparenting were higher in psychological distress. These findings align with prior research on helicopter and anxious overprotective parenting (Mathijs et al. 2024; Van Petegem et al. 2020). However, the association was significantly stronger for demanding overparenting and, when controlling for demanding overparenting, responsive overparenting was no longer significantly related to youth's distress. A recent meta‐analysis examining intrusive parenting similarly found that anxious overparenting was more strongly associated with higher levels of youth's internalizing and externalizing symptoms (*r* = 0.23 and *r* = 0.20, respectively) than was helicopter parenting (*r* = 0.18 and *r* = 0.11, respectively; Ryan et al. 2026). Future research could further differentiate specific adolescent symptoms in relation to both demanding and responsive overparenting.

## 8 Youth's Decision‐Making and Coping Self‐Efficacy

Consistent with past research on overprotective parenting and coping (Flamant et al. 2022; Odenweller et al. 2014), youth who reported experiencing a higher level of demanding and responsive overparenting also reported lower coping efficacy, although the association of responsive overparenting with coping efficacy was no longer significant after controlling for demanding overparenting. The perception of efficacy when coping with stress and access to many coping strategies are central to adolescents' resilience and capacity to manage developmental challenges (Modecki et al. 2017). As adolescents transition into emerging adulthood, they are expected to develop greater independence from their parents in coping with stress (Skinner and Zimmer‐Gembeck 2016). However, such development can be hindered when parents take over tasks for adolescents that allow them to avoid challenge or restrict self‐direction. This suggests that when youth perceive that their parents are generally higher in overparenting—for example, by taking over tasks from them that they feel they could do on their own—they are perhaps not having the experiences that develop a greater sense of coping efficacy.

Similar to the results for coping efficacy, demanding and responsive overparenting were also associated with some signs that adolescents were having difficulty with decision‐making. When controlling for the other form of overparenting, demanding overparenting—not responsive overparenting—was associated with less rational and more avoidant and impulsive decision‐making. Nevertheless, responsive overparenting was associated with adolescents' report of more dependent decision‐making, both before and after controlling for demanding overparenting. These findings suggest that demanding overparenting is most limiting for adolescents' development of general decision‐making skills, but that adolescents who report their parents are higher in responsive overparenting may be more reliant or dependent on others, including parents, when making decisions. Thus, although responsive overparenting appeared to be positive or at least not negative for youth, this does indicate that youth will feel more reliant on parents for decision‐making support when parents are more responsive and step in when their adolescent children seem to be facing challenge or stressors. Further research is needed to help clarify the mechanisms through which adolescents develop coping and decision‐making competencies in the context of overprotective parenting.

## 9 Limitations

There are three limitations of this study to highlight. First, the study had a cross‐sectional design. Although this design was sufficient for the study aims, it does not allow for causal inferences or the testing of directional effects. A longitudinal study will be needed to examine how demanding and responsive overparenting prospectively relate to parent and child outcomes, and vice versa. A second limitation is the sole reliance on youth report. Although reports from youth are important in studies of parenting and associated adjustment in youth, the observed associations could be partly attributable to shared method variance. Thus, differences in findings of this study compared to the previous study of demanding and responsive overparenting, which relied only on parent report (Ryan et al. 2024), may be partly due to shared method variance in both studies. Future research would benefitfrom collecting multi‐informant data. Third, there was no control for other factors that may explain outcomes beyond demanding and responsive overparenting. Future research could consider whether demographic and background variables account for demanding and responsive overparenting, as well as their associations with parent and youth outcomes.

## 10 Conclusion

The findings of this study are consistent with the idea that measures of overprotective and helicopter parenting may be better differentiated into a more coercive or controlling pattern of overparenting—referred to as demanding overparenting—from overparenting that may be more likely to appear as parents' excessive help or interference in their adolescent or emerging adult children's tasks and decision‐making, such as during times of stress—referred to as responsive overparenting. It may be that responsive overparenting, while still reflecting parental overinvolvement, is more contextually specific, with closer links to situations where the adolescent may seem to require adult help or additional support. Distinguishing demanding from responsive overparenting underscores the importance of considering nuanced parental motivations and behaviors when evaluating the effects of overprotectiveness or parental overinvolvement on youth development. Future research should continue to disentangle these features of overparenting and examine their longitudinal consequences, thereby informing interventions aimed at fostering adolescent autonomy, resilience, and healthy parent–child dynamics during adolescence and emerging adulthood.

## Author Contributions

**Melanie J. Zimmer‐Gembeck:** conceptualization, methodology, data curation, investigation, formal analysis, supervision, funding acquisition, project administration, writing – original draft, writing – review and editing. **Katherine M. Ryan:** conceptualization, data curation, project administration, writing – review and editing, methodology, investigation. **Bart Soenens:** conceptualization, funding acquisition, writing – review and editing, methodology. **Grégoire Zimmermann:** conceptualization, funding acquisition, writing – review and editing, methodology. **Stijn Van Petegem:** conceptualization, funding acquisition, writing – review and editing, methodology.

## Ethics Statement

The study was approved by Griffith University Human Research Ethics Committee (GU Ref No: 2022/436). The study was carried out in accordance with the World Medical Association Declaration of Helsinki.

## Consent

Informed consent to participate was written.

## Conflicts of Interest

The authors declare no conflicts of interest.

## Data Availability

The data are available upon request from the first author. The study reported in this article was not formally preregistered. Neither the data nor the materials have been made available on a permanent third‐party archive; requests for the data or materials can be sent via email to the lead author at m.zimmer-gembeck@griffith.edu.au.
